# Biological mechanisms of different exercise modalities in improving sleep disorders: A review

**DOI:** 10.1016/j.ibneur.2026.07.023

**Published:** 2026-08-02

**Authors:** Lingtao Zhao, Jingzhi Leng, Chunsheng Zhao, Juntao Cui

**Affiliations:** a Department of Biomedical Engineering, The Hong Kong Polytechnic University, 999077, Hong Kong, China; b College of Sports and Human Science, Key Laboratory of Sports and Physical Fitness Health of Ministry of Education, Beijing Sport University, Beijing 100091, China; c School of Physical Education, Qingdao University, Qingdao 266071, China; d School of Basic Medicine, Institute of Brain Science and Disease, Shandong Provincial Collaborative Innovation Center for Neurodegenerative Disorders, Shandong Key Laboratory of Pathogenesis and Prevention of Brain Diseases, Qingdao University, Qingdao 266071, China

**Keywords:** Sleep disorders, Physical activity, Aerobic exercise, Resistance training, Mind-body therapies

## Abstract

Sleep disorders such as insomnia, sleep apnea, and circadian rhythm dysfunctions constitute a major public health challenge due to their high prevalence and associated risks for physical and mental health. In response to the limitations and potential side effects of pharmacological treatments, non-pharmacological interventions have become increasingly central to management strategies. Among these, structured physical exercise has emerged as a particularly prominent and evidence-supported approach, valued for its demonstrated efficacy and favorable safety profile. This narrative review aims to consolidate and examine the contemporary body of scientific evidence elucidating how distinct forms of exercise, primarily categorized as aerobic endurance training, resistance or strength training, and mindful mind-body practices, act to alleviate various sleep disturbances. The underlying biological pathways through which these exercise modalities exert their positive effects are complex and interconnected, encompassing thermoregulatory processes, neurochemical adaptations, endocrine signaling, and shifts in autonomic nervous system balance.

## 1 Introduction

Sleep is not a passive state of rest but a highly sophisticated and dynamically regulated physiological process. It serves as a foundational pillar for systemic homeostasis, underpinning critical functions such as cellular repair, the clearance of metabolic waste products from the brain, and the rebalancing of the neurochemical milieu (Lange, 2026, Turi, 2025, Tomatsu et al., 2025). Its role in cognitive consolidation is equally vital through mechanisms like hippocampal-neocortical dialog and memory replay, sleep facilitates the transformation of short-term memories into long-term storage and integrates newly learned information (Ngo et al., 2020, Hahn, 2024). Furthermore, robust sleep is indispensable for preserving metabolic equilibrium by regulating key hormones such as insulin, leptin, and ghrelin, and for maintaining optimal immune competence as it orchestrates the nocturnal release of cytokines and supports adaptive immune memory (Ramasubbu, 2023, van Egmond, 2023, Namisaki et al., 2024, Spaeth, 2015). Given these core functions, sleep disorders represent a significant global public health burden associated with increased risks of cardiovascular disease, metabolic syndrome, neurodegenerative disorders, and psychiatric conditions.

The pathophysiology of chronic insomnia and obstructive sleep apnea is inherently complex and seldom arises from an isolated cause. Rather, it typically results from multifaceted dysregulation across several deeply interconnected biological systems, including disruption of the circadian timing network that desynchronizes the sleep-wake cycle from environmental cues like light and darkness, imbalances within key neuroendocrine axes exemplified by hypothalamic-pituitary-adrenal axis hyperactivity that disturbs cortisol rhythms and growth hormone secretion, and a shift in autonomic nervous system balance toward heightened sympathetic tone (Chaput, 2023, Tobaldini, 2017, Asarnow, 2020). The resulting state of persistent physiological hyperarousal, marked by elevated heart rate and cortisol, is fundamentally incompatible with the parasympathetic-dominant state necessary for restorative sleep. Moreover, chronic low-grade systemic inflammation and metabolic dysfunction are frequently intertwined with these disturbances, forming a self-perpetuating cycle (Duan, 2023, Rubio-Valles and Ramos-Jimenez, 2025).

Within this intricate etiological framework, structured physical exercise presents itself as a uniquely powerful non-pharmacological intervention. It acts as a broad-spectrum physiological modulator with the capacity to simultaneously and synergistically target the very pathways characteristically impaired in sleep pathologies, including strengthening circadian signals, regulating neuroendocrine responses, improving autonomic balance, attenuating systemic inflammation, and optimizing metabolic function (Baranwal et al., 2023, Takemura, 2024, Mahalakshmi, 2020, Sato, 2021, Acosta-Manzano, 2022). This multi-targeted mechanism of action makes exercise an ideal strategy for addressing the multifactorial nature of sleep disorders.

While the benefits of exercise for sleep are widely observed, the biological mechanisms through which different modalities, primarily aerobic endurance training, resistance training, and mind-body practices that integrate focus, breath control, and meditation, exert their effects show both overlap and distinct emphasis. Understanding these specificities and commonalities is crucial for developing precise personalized exercise prescriptions. Therefore, the central aim of this narrative review is to examine, analyze, and synthesize current scientific evidence to elucidate the distinct and overlapping biological mechanisms by which the primary categories of exercise improve sleep. Specifically, it will explore how aerobic exercise, resistance training, and mind-body practices respectively impact thermoregulatory, neurochemical, endocrine, metabolic, and autonomic pathways to produce objective enhancements in total sleep time, slow-wave sleep proportion, and sleep spindle characteristics, as well as subjective improvements in sleep quality, depth, and restorative value. Through this mechanistic dissection, we aim to provide a robust theoretical foundation and more targeted guidance for the non-pharmacological treatment of sleep disorders based on exercise modality.

## 2 Methodological

For this narrative review, we conducted a comprehensive literature search using the PubMed, Web of Science, and Scopus electronic databases. The search covered articles published primarily between January 2015 and February 2026, although seminal earlier works were also included when necessary for historical context or foundational concepts. Search keywords comprised combinations of terms related to sleep disorders such as insomnia, sleep apnea, sleep quality, circadian rhythm, and sleep disturbance combined with exercise modalities including physical activity, aerobic exercise, resistance training, strength training, mind-body therapy, yoga, Tai Chi, and Qigong along with biological mechanism terms such as thermoregulation, neurochemistry, endocrine, autonomic nervous system, inflammation, and brain-derived neurotrophic factor. We selected peer-reviewed original research articles, systematic reviews, and meta-analyses that investigated the biological mechanisms through which structured exercise influences sleep outcomes in adult human populations. Studies focusing exclusively on pediatric populations or those lacking mechanistic insight were excluded. Due to the narrative nature of this review, a formal risk-of-bias assessment tool was not applied however the strength of evidence from different study designs including randomized controlled trials, systematic reviews, meta-analyses, animal studies, and observational studies is discussed qualitatively throughout the manuscript to help readers evaluate the robustness of conclusions. This methodological approach ensures transparency and allows readers to assess the scope and limitations of the evidence synthesized in this review.

## 3 Aerobic exercise

Aerobic exercise, encompassing activities such as running, cycling, and swimming, represents the most extensively researched exercise modality for enhancing sleep (Vlahoyiannis, 2021, de Almeida, 2025, Kovacevic, 2018). Its beneficial effects are mediated through a convergence of interconnected physiological mechanisms. The thermoregulatory hypothesis provides a foundational model wherein prolonged aerobic activity induces a significant rise in core body temperature. The subsequent post-exercise decline in temperature is not merely a return to baseline but is thought to actively facilitate sleep onset by amplifying the body's natural nocturnal temperature drop. This exaggerated cooling signal is detected by the hypothalamus, a key sleep-regulating center, and is strongly coupled to increased sleep propensity, potentially leading to a more rapid transition into sleep and an enhancement in both the duration and restorative quality of slow-wave sleep (Di Cristoforo, 2015, Latifi, 2018). Evidence supporting this mechanism comes primarily from randomized controlled trials demonstrating consistent associations between exercise-induced temperature elevation and subsequent sleep depth.

Concurrently, aerobic exercise induces significant neurochemical modulation within the brain. It promotes the upregulation of brain-derived neurotrophic factor, a protein crucial for neuronal health, survival, and plasticity (de Matos, 2025). This effect is particularly important in brain regions governing sleep-wake cycles, such as the hypothalamus and brainstem nuclei, where brain-derived neurotrophic factor may help optimize neural circuitry for sleep regulation (Veronese, 2019), although much of this evidence derives from animal models and requires further human validation. Furthermore, aerobic activity alters the dynamics of key monoamine neurotransmitters, including serotonin and norepinephrine, which are intimately involved in arousal, mood, and sleep stages (de Lemos Muller, 2023, Zhao, 2025). Perhaps most directly, sustained aerobic exercise increases the accumulation of adenosine, a neuromodulator whose rising levels in the basal forebrain correlate with increased homeostatic sleep drive and exert a natural soporific effect (Twycross-Lewis, 2016, Całkosiński, 2021).

**Fig. 1:**
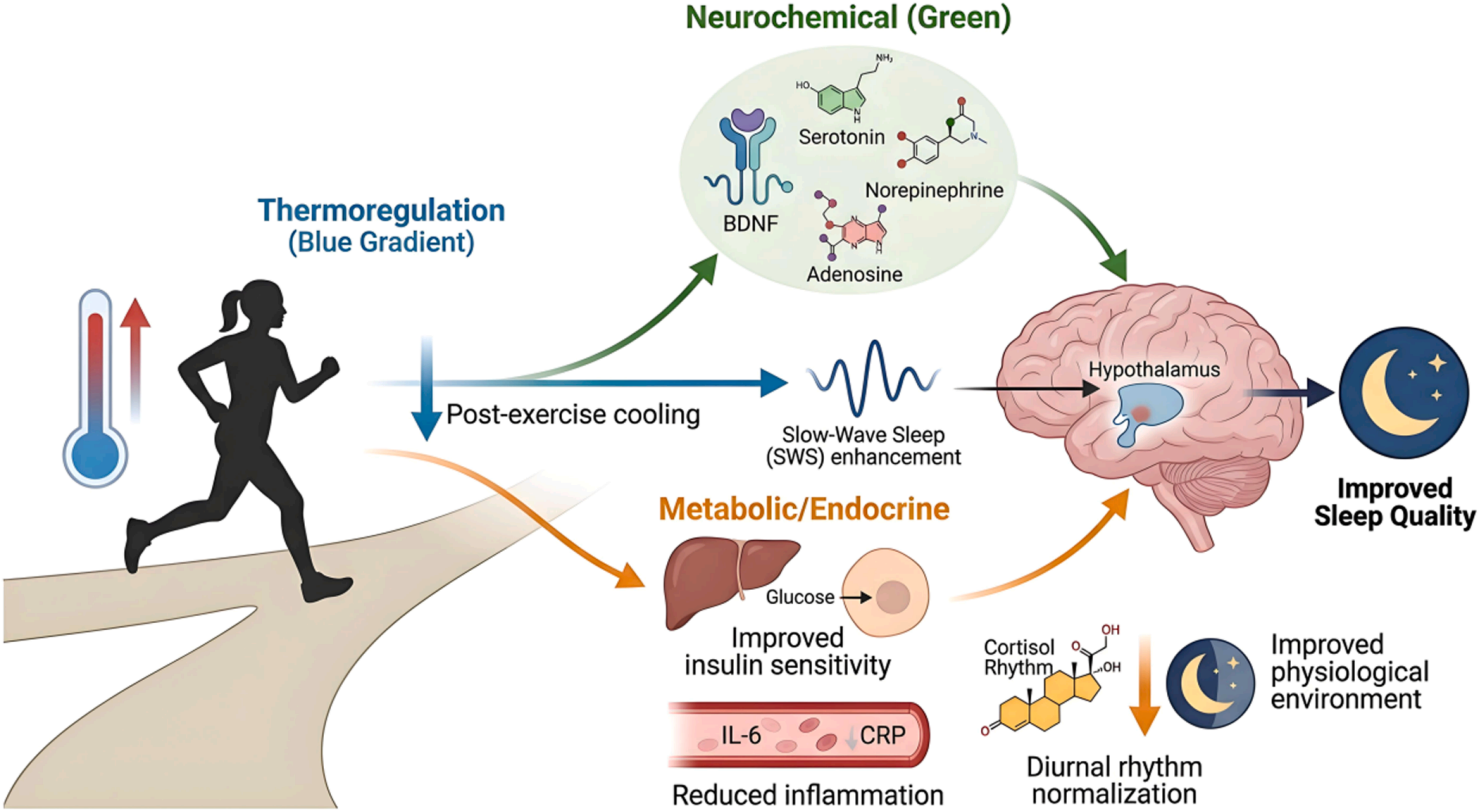
Schematic illustration of the integrative physiological mechanisms by which aerobic exercise promotes sleep. The central runner silhouette signifies the initiation of aerobic activity, triggering three interconnected systemic pathways. The thermoregulatory pathway depicts the characteristic post-exercise decline in core body temperature, which acts as a physiological signal to the hypothalamus to facilitate sleep onset and augment slow-wave sleep (SWS). The neurochemical pathway illustrates the upregulation of key neuromodulators including brain-derived neurotrophic factor (BDNF), serotonin, norepinephrine, and adenosine that collectively suppress arousal, enhance neural plasticity, and reinforce homeostatic sleep drive. Concurrently, the metabolic/endocrine pathway encompasses broad systemic benefits, such as enhanced insulin sensitivity and glucose metabolism, attenuation of inflammatory biomarkers interleukin−6 and C-reactive protein, and the restoration of circadian cortisol rhythms. These multisystem adaptations converge to establish a physiological environment conducive to improved sleep quality, characterized by reduced sleep latency, consolidated sleep architecture, and enhanced restorative SWS.

Beyond these direct neural and thermal effects, regular aerobic exercise confers profound endocrine and metabolic benefits that indirectly promote healthier sleep. It significantly improves insulin sensitivity and glucose metabolism while reducing chronic low-grade systemic inflammation, as evidenced by lower circulating levels of markers such as C-reactive protein and interleukin−6, findings consistently reported in large-scale meta-analyses (Kim et al., 2025, Abd El-Kader and Al-Jiffri, 2019). Given that both metabolic dysfunction and inflammation are established correlates and contributors to fragmented and poor-quality sleep, their amelioration through exercise creates a more hospitable physiological environment for sleep. Additionally, consistent aerobic training helps normalize the diurnal rhythm of cortisol, the primary stress hormone, promoting a steeper and more pronounced decline in the evening as demonstrated by randomized controlled trials (De Nys, 2022). This attenuated evening cortisol level reduces physiological arousal and is highly conducive to relaxed sleep initiation as illustrated in Fig. 1.

## 4 Resistance training

Resistance training, including weightlifting and bodyweight exercises, exerts a positive influence on sleep through several distinct and complementary biological pathways. A primary mechanism involves its potent effect on endocrine signaling, particularly the secretion of growth hormone. High-intensity resistance exercise is a powerful stimulator of growth hormone release as demonstrated by randomized controlled trials (Athanasiou et al., 2023). Given that the majority of physiological growth hormone secretion occurs during periods of slow-wave sleep, a compelling synergistic relationship is established. The exercise-induced surge in growth hormone may effectively create a heightened physiological demand for the deep restorative state of slow-wave sleep, thereby potentially increasing its proportion and consolidating its architecture (Kredlow, 2015), although it should be noted that this association remains largely correlational and direct causal evidence from human studies linking exercise-induced growth hormone pulses to subsequent slow-wave sleep augmentation is still limited. This process is believed to support the anabolic repair and recovery of musculoskeletal tissues stressed during training, linking daytime activity directly to nocturnal restorative processes.

Furthermore, resistance training induces significant beneficial changes in metabolism and body composition that secondarily foster better sleep. By increasing lean muscle mass, it elevates basal metabolic rate and improves overall metabolic health including insulin sensitivity as supported by systematic reviews and meta-analyses (de Souza, 2017). This enhancement of metabolic profile is particularly relevant for sleep-related breathing disorders; in individuals with obesity, improved body composition and metabolic function can reduce the severity of obstructive sleep apnea by decreasing upper airway collapsibility and systemic inflammation (Lin, 2024). More broadly, by mitigating the metabolic stress and dysregulation that are known to disrupt sleep continuity and architecture, resistance training helps create a more stable internal environment conducive to sustained sleep.

**Fig. 2:**
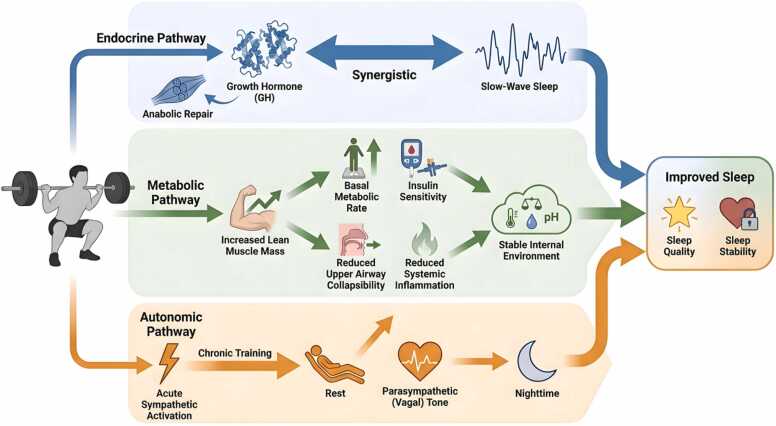
Integrated mechanistic pathways linking resistance training to improved sleep quality and stability. illustrates the potent post-exercise surge in growth hormone (GH), which exhibits a synergistic relationship with slow-wave sleep; this reciprocal interaction facilitates anabolic tissue repair while promoting deep, restorative sleep stages. The metabolic pathway depicts increases in lean muscle mass and basal metabolic rate, driving enhanced insulin sensitivity and contributing to a stable internal physiological environment; concurrently, these adaptations reduce upper airway collapsibility and mitigate systemic inflammation, thereby diminishing sleep-disruptive arousals. The autonomic nervous system pathway demonstrates the biphasic response to training, transitioning from acute sympathetic activation during the lifting bout to a chronic enhancement of parasympathetic tone during rest and nighttime, which stabilizes cardiovascular and respiratory functions. Collectively, these interconnected adaptations orchestrate a systemic environment conducive to both consolidated sleep architecture and overall sleep stability.

Additionally, while a single session of resistance training acutely activates the sympathetic nervous system, chronic regular practice promotes a favorable long-term adaptation in autonomic nervous system balance as evidenced by randomized controlled trials and longitudinal observational studies (Medellín Ruiz, 2025, Andreu-Caravaca, 2022). Over time, it enhances parasympathetic vagal tone, particularly during post-exercise recovery periods and at rest. This shift toward greater parasympathetic dominance is crucial for sleep, as increased vagal activity during the nighttime is strongly associated with improved sleep quality, greater cardiac stability, and more rapid sleep onset. Thus, through consistent practice, resistance training helps lower pervasive physiological arousal and fosters a state of calm that is foundational for initiating and maintaining sleep as illustrated in Fig. 2.

## 5 Mind-body therapies

Mind-body therapies, such as Yoga, Tai Chi, and Qigong, represent a unique category of intervention that integrates mild to moderate physical activity with deliberate focus, controlled breathing, and meditative awareness. This synthesis of components targets sleep disturbances primarily by countering the hyperarousal, both physiological and cognitive, that is central to conditions like insomnia (Saadh, 2026). The primary mechanism of action is the profound regulation of the autonomic nervous system and the hypothalamic-pituitary-adrenal axis, the body's core stress response system, as demonstrated by randomized controlled trials (Lee, 2025). Through deliberate breath control and gentle movement, these practices consistently promote a shift toward parasympathetic dominance, reducing sympathetic fight-or-flight activity and lowering baseline physiological arousal. Concurrently, they dampen the reactivity of the hypothalamic-pituitary-adrenal axis, leading to more regulated and often reduced cortisol levels, which is particularly critical for individuals whose insomnia is driven or exacerbated by stress (Moraes, 2018).

Emerging neurobiological evidence points to a complementary mechanism involving the brain's primary inhibitory neurotransmitter system. Preliminary research, primarily from animal studies and small-scale human investigations, suggests that regular engagement in mind-body practices may increase the activity and availability of gamma-aminobutyric acid (Krishnakumar et al., 2015). As low GABAergic tone is strongly associated with a state of neuronal hyperarousal and insomnia, this potential upregulation provides a neurochemical foundation for the increased calm and reduced anxiety reported by practitioners, although these findings warrant cautious interpretation until confirmed by larger clinical trials.

**Fig. 3:**
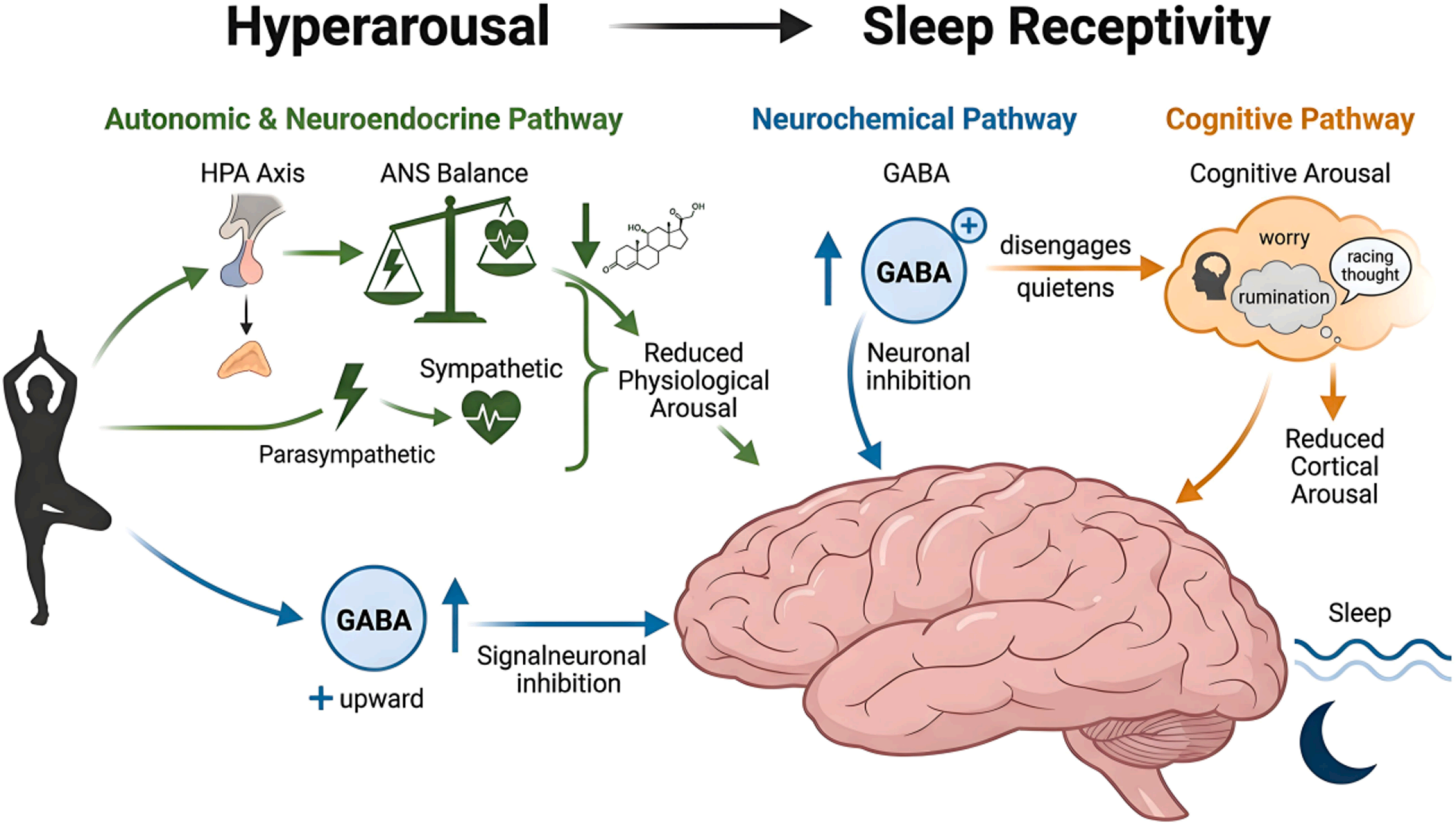
Mechanistic illustration of the transition from physiological and cognitive hyperarousal to sleep receptivity mediated by mind-body therapies. The silhouette engaging in meditative practice initiates a cascade of multisystem adaptations. The autonomic and neuroendocrine pathway illustrates the suppression of stress response systems, characterized by a shift in autonomic balance toward parasympathetic dominance and attenuated HPA axis activity, effectively dampening physiological arousal. Concurrently, the neurochemical pathway highlights the upregulation of the inhibitory neurotransmitter GABA, which facilitates neuronal inhibition and promotes a state of central nervous system calm. Complementing these, the cognitive pathway depicts the disengagement from maladaptive thought patterns such as worry and rumination thereby alleviating cortical hyperarousal. Collectively, these integrated physiological and psychological adjustments attenuate global hyperarousal, effectively bridging the gap between wakefulness and the onset of restorative sleep.

Furthermore, these exercises directly target cognitive and cortical arousal, a key perpetuating factor in chronic sleep disorders. By cultivating a present-focused non-judgmental awareness, they train individuals to disengage from the pre-sleep cognitive rumination, worry, and racing thoughts that inhibit sleep onset. This practice of cognitive deactivation helps quiet the cortex, facilitating the transition from a state of active thinking to one receptive to sleep as supported by observational studies and neuroimaging research (Yu, 2018, Chen, 2020). Thus, through integrated effects on the autonomic, neuroendocrine, neurochemical, and cognitive systems, mind-body therapies address the multifaceted nature of arousal that disrupts sleep as illustrated in Fig. 3.

## 6 Overlapping and comparative mechanisms

While aerobic exercise, resistance training, and mind-body practices engage distinct primary pathways to improve sleep, they also share several fundamental overlapping mechanisms that collectively contribute to better sleep regulation. A principal common pathway is the consolidation of circadian rhythms. Daily physical activity, particularly when performed at a consistent time of day, acts as a potent zeitgeber that helps synchronize the central circadian clock in the suprachiasmatic nucleus, with this effect being well documented across observational studies and controlled trials. This reinforcement of the circadian timing system strengthens the sleep-wake cycle, promoting more stable and predictable sleep patterns. Furthermore, all forms of exercise are well-established mediators of mood, effectively reducing symptoms of depression and anxiety as demonstrated by numerous systematic reviews and meta-analyses. Since dysregulated mood is a major contributor to sleep initiation and maintenance problems, this psychophysiological benefit serves as a significant cross-cutting mediator of improved sleep across all modalities. From a neurobiological perspective, these modalities also converge on the energy restoration theory. Exercise, regardless of type, depletes hepatic glycogen and increases adenosine triphosphate turnover, elevating the homeostatic sleep drive. This is reflected in the accumulation of sleep-promoting substances like adenosine in the basal forebrain, thereby creating a stronger physiological pressure for sleep and deepening subsequent slow-wave sleep for bodily restoration.

**Fig. 4:**
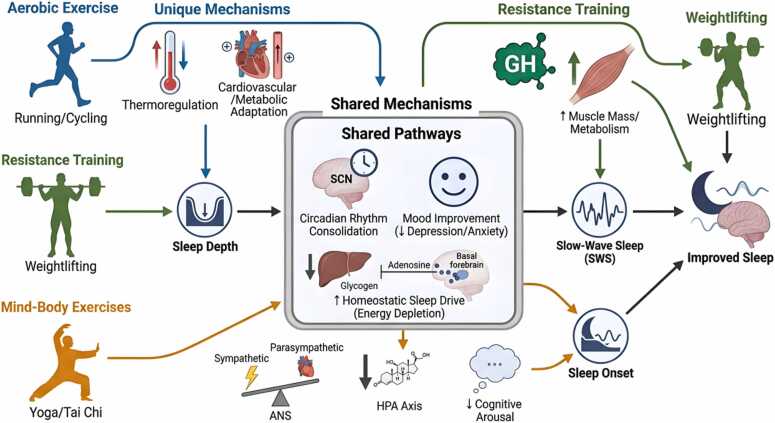
Integrative schema delineating the shared and modality-specific mechanisms underlying the sleep-enhancing effects of exercise. The central core illustrates universal pathways conserved across all exercise modalities, which are essential for fundamental sleep regulation. These shared mechanisms include the entrainment of circadian rhythms, mood improvement, and the augmentation of homeostatic sleep drive. Radiating outward, the schema details distinct adaptive responses specific to each modality that synergize with the common core to produce unique therapeutic outcomes. Aerobic exercise primarily leverages thermoregulatory and cardiovascular-metabolic adaptations to deepen overall sleep architecture. Resistance training predominantly stimulates anabolic processes specifically growth hormone release and enhanced muscle metabolism to potentiate SWS. Conversely, mind-body therapies specialize in autonomic neuromodulation and the attenuation of psychological hyperarousal, thereby facilitating rapid sleep onset. This comprehensive model underscores how the synergistic interplay between shared systemic effects and specific physiological specializations defines the efficacy of a multi-modal exercise approach for optimizing sleep quality.

However, the emphasis and relative potency of these mechanisms differ notably among exercise types, leading to their unique therapeutic profiles. Aerobic exercise exerts its most pronounced effects through robust thermoregulatory adaptations and systemic cardiovascular and metabolic endurance adaptations, making it particularly effective for enhancing sleep depth through temperature-driven processes and improving sleep in conditions linked to metabolic dysfunction. In contrast, resistance training specializes in targeting anabolic hormone release, most notably growth hormone, and fundamentally altering muscle metabolism and body composition, making it uniquely suited to increase the proportion of restorative slow-wave sleep and address sleep issues secondary to sarcopenia or metabolic syndrome, although it should be noted that the direct causal link between resistance training-induced growth hormone secretion and subsequent slow-wave sleep enhancement requires further investigation through controlled human trials. Mind-body therapies, while involving mild physical activity, excel most prominently in the direct modulation of psychophysiological arousal. Their core strength lies in down-regulating the stress response systems, namely the sympathetic nervous system and the hypothalamic-pituitary-adrenal axis, and cultivating cognitive detachment from ruminative thought patterns, offering a targeted intervention for the hyperarousal that characterizes conditions like stress-related insomnia. Therefore, the overall sleep benefit of any exercise regimen likely arises from a combination of these universal effects and the specific modality-dependent actions on particular physiological and psychological systems as summarized in Table 1 and illustrated in Fig. 4.

**Table 1:** Summary of the physiological mechanisms and primary sleep benefits associated with different exercise modalities.

**Exercise modality**	**Main mechanisms**	**Primary sleep benefits**
Aerobic Exercise	Thermoregulation post-exercise core temperature decline; Neurochemical upregulation of BDNF and adenosine; Endocrine reduction of evening cortisol; Metabolic improvement in insulin sensitivity and inflammation	Faster sleep onset; Increased slow-wave sleep duration; Improved sleep efficiency; Reduced sleep fragmentation
Resistance Training	Endocrine stimulation of growth hormone; Metabolic increase in lean muscle mass and basal metabolic rate; Autonomic shift toward long-term parasympathetic dominance	Increased proportion of restorative slow-wave sleep; Improved sleep stability; Reduced severity of obstructive sleep apnea
Mind-Body Therapies	Autonomic promotion of parasympathetic dominance; Neuroendocrine dampening of HPA axis activity and cortisol; Neurochemical potential upregulation of GABA; Cognitive reduction of rumination and cortical arousal	Reduced sleep onset latency; Decreased wake after sleep onset; Improved subjective sleep quality; Effective for stress-related insomnia

## 7 Expand clinical implications

The mechanistic insights provided by this review carry direct translational value for clinical practice and exercise prescription. For clinicians and sleep specialists, the distinct biological profiles of each exercise modality suggest that treatment selection should be guided by the patient's predominant sleep disorder pathophysiology rather than a one-size-fits-all approach. For patients presenting with stress-related insomnia characterized by physiological and cognitive hyperarousal, mind-body therapies such as yoga or Tai Chi should be considered as first-line exercise interventions due to their direct effects on autonomic balance, hypothalamic-pituitary-adrenal axis regulation, and cognitive deactivation as supported by randomized controlled trials (Saadh, 2026, Lee, 2025, Moraes, 2018). For individuals with metabolic comorbidities such as type 2 diabetes or obesity who also suffer from poor sleep quality, aerobic exercise is particularly indicated because of its robust effects on thermoregulation, insulin sensitivity, and systemic inflammation reduction (Kim et al., 2025, Abd El-Kader and Al-Jiffri, 2019, De Nys, 2022). For older adults with sarcopenia or patients with sleep-disordered breathing, resistance training offers unique benefits through growth hormone stimulation, increased lean muscle mass, and improved upper airway stability (Athanasiou et al., 2023, Kredlow, 2015, de Souza, 2017, Lin, 2024). For exercise professionals designing community or clinical programs, an integrated multimodal regimen combining elements of all three exercise types is recommended to achieve comprehensive mechanistic coverage, such as a weekly schedule incorporating 150 min of moderate-intensity aerobic activity, two sessions of resistance training targeting major muscle groups, and one to two sessions of mind-body practice emphasizing breath control and meditative awareness. Timing considerations are also clinically relevant; vigorous aerobic exercise should generally be completed at least two to three hours before bedtime to allow core body temperature to decline adequately, whereas mind-body practices may be safely performed closer to sleep onset due to their calming effects. For rehabilitation practitioners working with patients who have comorbid medical conditions such as heart failure, chronic pain, or mobility limitations, a graduated approach is advisable beginning with low-impact mind-body therapies to establish tolerance and improve autonomic regulation, followed by progressive introduction of adapted aerobic and resistance exercises as functional capacity improves. Throughout all clinical applications, sleep quality should be monitored as a vital sign of recovery and exercise tolerance, allowing for dynamic adjustment of prescription parameters including intensity, duration, frequency, and modality selection based on individual response. These practical recommendations bridge the gap between mechanistic understanding and real-world application, empowering healthcare professionals to leverage exercise as a targeted non-pharmacological tool for sleep improvement.

## 8 Limitations

Despite the comprehensive synthesis of evidence presented in this review, several important limitations of the current literature must be acknowledged to provide appropriate context for interpreting our conclusions. Considerable heterogeneity exists across studies in terms of exercise protocols, including variations in intensity, duration, frequency, modality specificity, and timing relative to the sleep period, which makes direct comparisons between studies and across exercise modalities challenging and limits the ability to prescribe precise exercise doses for optimal sleep benefits. A substantial proportion of clinical trials rely on subjective sleep measures such as the Pittsburgh Sleep Quality Index or sleep diaries rather than objective polysomnography or actigraphy, which restricts our ability to confirm specific changes in sleep architecture including slow-wave sleep proportion, sleep spindle characteristics, and sleep continuity parameters that are critical for understanding mechanistic pathways. The duration of most intervention studies is relatively short, typically ranging from four to sixteen weeks, leaving unanswered questions about long-term adherence to exercise regimens and the sustainability of sleep improvements beyond the intervention period. There is a notable imbalance in the volume of research across the three exercise modalities, with aerobic exercise receiving disproportionately more investigation than resistance training or mind-body therapies, which may skew the apparent strength of evidence and create an incomplete picture of comparative effectiveness. Publication bias toward positive findings is a recognized concern across the exercise-sleep literature, as studies reporting null or negative results are less likely to be published, potentially overestimating the true magnitude of exercise benefits on sleep outcomes. The generalizability of current evidence is limited because most studies have been conducted in healthy young to middle-aged adults or specific clinical populations such as individuals with insomnia or obstructive sleep apnea, with insufficient representation of children, adolescents, older adults above 75 years, and diverse ethnic and socioeconomic backgrounds. The interplay between exercise and other lifestyle factors that influence sleep, including dietary habits, caffeine and alcohol consumption, light exposure patterns, psychosocial stress, and screen time before bed, is rarely controlled for in existing studies, making it difficult to isolate the independent effects of exercise from confounding variables. Many mechanistic studies, particularly those examining neurochemical and neuroendocrine pathways, have been conducted in animal models, and direct translation of these findings to human sleep physiology requires cautious interpretation until confirmed by human experimental research. By explicitly acknowledging these limitations, we aim to provide a balanced perspective that informs the interpretation of current evidence and guides future research efforts toward addressing these critical gaps.

## 9 Conclusion and future directions

In conclusion, the collective evidence presented in this narrative review underscores that diverse exercise modalities, including aerobic endurance training, resistance training, and mind-body practices, confer significant benefits for sleep health by engaging a confluence of interconnected biological mechanisms. These mechanisms strategically target the core physiological systems often dysregulated in sleep disorders, encompassing thermoregulatory pathways, neurochemical balance, endocrine signaling, metabolic function, and autonomic nervous system tone. Each modality exhibits a distinct mechanistic profile that allows for targeted physiological effects. Aerobic exercise demonstrates particular potency in modulating thermoregulatory processes and reducing systemic inflammation, with strong support from randomized controlled trials and meta-analyses (Kim et al., 2025, Abd El-Kader and Al-Jiffri, 2019, De Nys, 2022). Resistance training is highly effective in stimulating anabolic hormone release and altering body composition, although the direct causal link between growth hormone secretion and slow-wave sleep enhancement requires further human experimental confirmation (Athanasiou et al., 2023, Kredlow, 2015, de Souza, 2017, Lin, 2024). Mind-body therapies excel in regulating autonomic balance and dampening hyperactivity of the hypothalamic-pituitary-adrenal axis, with evidence primarily derived from randomized controlled trials and observational studies (Saadh, 2026, Lee, 2025, Moraes, 2018). Given this complementary nature, an integrated exercise regimen that combines elements of these different modalities may offer the most comprehensive mechanistic coverage, thereby providing a robust multi-system intervention for improving both sleep architecture and subjective sleep quality.

Looking forward, several critical avenues for future research emerge from the limitations identified in the current literature. First, there is a pressing need for more direct head-to-head comparative effectiveness trials that rigorously assess different exercise modalities against each other and against standard care for specific sleep disorder subtypes, ideally employing objective polysomnographic outcomes alongside subjective measures. Second, research must move toward precisely defining the optimal dose of exercise for sleep, including the prescription of specific parameters for intensity, duration, frequency, and importantly timing relative to the sleep period, as this can influence circadian phase and core body temperature dynamics in ways that may either enhance or impair sleep depending on individual chronotype. Third, longer-term intervention studies with extended follow-up periods are needed to evaluate the sustainability of sleep improvements and adherence to exercise regimens beyond initial intervention phases. Fourth, future studies should strive for greater diversity in study populations to enhance generalizability across age groups, ethnicities, and clinical conditions. Finally, and perhaps most pivotally, the field must advance toward personalized exercise prescriptions. This requires a deeper understanding of how an individual's specific sleep disorder pathophysiology, genetic background, chronotype, and comorbid conditions predict their response to a given exercise type. By integrating pathophysiology with precision in exercise prescription, future interventions can be tailored to maximize therapeutic efficacy for improving sleep, moving the field beyond generic recommendations toward individualized non-pharmacological sleep medicine.

## CRediT authorship contribution statement

**Jingzhi Leng:** Writing – review & editing, Investigation. **Chunsheng Zhao:** Writing – review & editing, Supervision, Conceptualization. **Juntao Cui:** Writing – review & editing, Supervision, Conceptualization. **Lingtao Zhao:** Writing – original draft, Data curation, Conceptualization.

## Declaration of Competing Interest

The authors declare that they have no ﬁnancial disclosures or conﬂicts of interest concerning the research related to this article.
